# The Influence of Viscosity and Non-freezing Water Contents Bounded to Different Hydroxypropyl Celluloses (HPC) and Hydroxypropyl Methylcelluloses (HPMC) on Stability of Acetylsalicylic Acid

**DOI:** 10.1208/s12249-019-1406-z

**Published:** 2019-05-15

**Authors:** Przemysław Talik, Joanna Piotrowska, Urszula Hubicka

**Affiliations:** Department of Inorganic and Analytical Chemistry, Pharmaceutical Faculty, Medical College, Jagiellonian University, 9 Medyczna St., 30-688 Krakow, Poland

**Keywords:** DSC, differential scanning calorimetry, non-freezing water, hydration of hydroxypropyl cellulose, hydration of hydroxypropyl methylcellulose

## Abstract

The aim of the study was to examine the influence of non-freezing water (NFW) contents bound to hydroxypropyl methylcellulose (HPMC) or hydroxypropyl cellulose (HPC) binary mixtures using acetylsalicylic acid (ASA) as a model moisture-sensitive ingredient. Polysaccharides with significantly different physicochemical properties were mixed with acetylsalicylic acid at a ratio 1:1 (*w/w*). The measurements of NFW contents of hydrated samples were carried out using differential scanning calorimetry (DSC). In the method used, the dry mass normalized dependency of melting enthalpy (ΔH) and respective contents of water was found to be linear. NFW values were calculated after extrapolation ΔH to 0. For stability studies, HPC/ASA and HPMC/ASA mixtures were stored at 40°C and 75% RH for 5 weeks in the climatic chamber. The ASA hydrolysis was investigated using UV-Vis spectrophotometry. The amounts of NFW calculated for raw HPMC 3 cP and 100,000 cP were 0.49 and 0.42 g g^−1^, while for polymer and ASA mixtures, prepared from HPC type LF (126 cP) and MF (6300 cP) as well as from HPMC 3 cP and 100,000 cP were 0.23, 0.28 g g^−1^, 0.21 g g^−1^, and 0.33 g g^−1^ respectively. The measured NFW values were connected with appropriate concentrations of unhydrolyzed ASA.

## INTRODUCTION

Chemical stability is an important factor of the drug quality because it is directly related to purity, efficacy, and safety of pharmacotherapy. In most drugs, the stability of a medicinal product as a whole depends not only on the properties of the active pharmaceutical ingredient (API), but also on the formulation and/or method of manufacture. One of the most important concepts in drug design is understanding the intrinsic stability of the API molecule and its degradation pathways. The most frequently encountered mechanism of drug degradation is hydrolysis, oxidation, and to some extent, photolysis (1). However, in multi-component heterogeneous systems, the degradation of the API can be more complex and may be the result of more than one factor – for example, hydrolysis with further oxidation, or *vice versa* [1]. It can be also possible that interactions between API molecules and excipients may be non-specific and thus less understandable. For that reason, drug stability is a constant interest of many research teams [2–6].

Both of hydroxypropyl cellulose (HPC) and hydroxypropyl methylcellulose (HPMC) are water-soluble derivatives of cellulose. They show a semi-crystalline structure, with a high degree of amorphous content and high molecular mobility [7]. These polysaccharides are nontoxic and biocompatible with human tissues [8]. Because their physical and chemical properties are simple to modify by synthesis, they are common dietary ingredients, can be used in surgical applications, and are one of the most important hydrophilic carrier materials used for the formulation of oral controlled drug delivery systems [9, 10]. The characteristic feature of these polymers is their ability to form solutions with various viscosity that depends on the size and the conformation of the polymer.

Interactions between water molecules and various carbohydrate polymers strongly modify both the properties of water itself and spatial structures of the polymer. Diversity of water behavior can be associated with capillary condensation effect [11], presence of “nanocavities” [12], that are able to trap water clusters, or strong interactions with polar groups of the polymer. Such interactions may be the result of direct binding to a chain (hydrogen bonding) or *via* another water molecule. The system of water and cellulose chains can be associated with the presence of two distinct fractions of water: bound water BW and free water FW. Additionally, bound water can be subcategorized to non-freezing water NFW and freezing bound water FBW [13]. The NFW is a fraction, where the first-order transitions, such as melting or freezing, are not calorimetrically observed. It does not crystallize even at − 100°C [14]. For low concentrations, all water is considered to be non-freezing [15]. However, still, there are no experimental and/or theoretical confirmations that the amount of non-freezing water is quantitatively the same water that is associated with polymer chains. Water that is less closely associated with polymer chains and crystallizes at a temperature below 0°C is called freezing bound water. Compared to free water FW, which crystallizes at 0°C, the FBW fraction has a significantly smaller melting enthalpy.

The aim of this work was to investigate the influence of viscosity as well as the non-freezing NFW water content on the stability of acetylsalicylic acid. Acetylsalicylic acid was chosen for these studies because it is the most commonly used hydrolyzable drug in a model of hygroscopic tablet formulations [16, 17]. Due to a large variation of semi-synthetic celluloses in terms of both molecular weight and viscosity, hydroxypropyl celluloses (HPC), and hydroxypropyl methylcelluloses (HPMC) were used for further investigations. The amounts of strongly associated NFW water, in both of raw polysaccharides or their 1:1 (*w/w*) mixtures with acetylsalicylic acid, were investigated using differential scanning calorimetry (DSC) method that was previously applied by other authors [18–21]. A decrease in the ASA content, accompanied by a proportional increase of salicylic acid (SA) content, was examined by means of UV-Vis spectrophotometry [22]. In summary, investigating the relationship between the content of non-freezing water and viscosity on the stability of API, may be crucial to better understanding the dissolution mechanisms and drug release of tablet formulations. The studied relationships have not been the subject to publication so far.

## EXPERIMENTAL

### Materials

The hydroxypropylmethylcelluloses (HPMC) of substitution type 2910 (Methocel E3 LV) and 2208 (Methocel K100 M), of viscosity 3 mPa s and 100,000 mPa s respectively, were provided by Dow Chemical (Michigan, USA). In the nomenclature of HPMC grades, an initial letters K and E identifies contents of methoxy groups of about 19–24% and 28–30% respectively. The number following the letter identifies the viscosities of that grade measured at 2% concentration in water at 20°C. The letter “M” is used to represent 1000 while “LV” refers to low viscosity. Investigated Klucel® Pharm hydroxypropyl celluloses types LF, MF, and HF were kindly provided by Hercules Incorporated, Aqualon Division. The physicochemical properties of polymers under study are presented in Table I.

**Table I:** Physicochemical properties of Klucel® Pharm hydroxypropyl celluloses (HPC) LF, MF, and hydroxypropyl methylcelluloses (HPMC) Methocels E3 LV and K100M

HPMC type	Type of substitution	Methoxy groups [%]	Hydroxypropoxy groups [%]	Viscosity [cP] (at solution concentration %)
Methocel E3 LV	2910	28.0–30.0	7–12	3 (2%)
Methocel K100M	2208	19.0–24.0	7–12	100,000 (2%)
HPC type	Molecular mass	Moles of substitution	Hydroxypropoxy groups [%]	Viscosity [cP] (at solution concentration %)
LF	95,000	3.6	72.8	126 (5%)
MF	850,000	3.8	74.8	6300 (2%)

Acetonitrile AR (POCh Gliwice) and formic acid AR (J.T. Baker) were analytical grade and commercially available.

## SAMPLE PREPARATION

### DSC Measurements

Both HPMC and HPC polymers as well as salicylate derivatives were kept in a vacuum desiccator over phosphorus pentoxide P_2_O_5_ for about 4 weeks. Appropriate physical mixtures were prepared after short (5 min) and gentle milling in agate mortar using a pestle. In this way, a 1:1 (*w/w*) mixtures of polysaccharides and salicylates were obtained. In order to obtain calibration curves, the dried 20–30 mg samples (16–18 samples) were accurately weighed, placed into plastic pans and an excess of Milli-Q water was added. To get the right concentration, the water was left to evaporate slowly at room temperature. In these studies, water content was expressed as *W*_*c*_, which is defined as H_2_O fraction related to the dry mass of raw polymers or their mixtures with ASA:

1 $$ {W}_C=\frac{m_{\mathrm{water}}}{m_{\mathrm{dry}}}\ \left[g\ {g}^{-1}\right] $$

In the DSC measurements, the *W*_*c*_ was ranging from 0.3 up to 3 g g^−1^. Next, the homogeneous material was immediately transferred and hermetically sealed. In order to obtain an equilibrium state, all sample pans were conditioned at room temperature for about 30 h. To protect from spontaneous reactions with water, before placing samples, all aluminum crucibles were exposed to steam in an autoclave to form a passive coat of Al_2_O_3_ (120°C for 3 h).

### ASA Stability Studies

Five grams of HPC/ASA or HPMC/ASA samples were scattered on Petri dishes and placed without covering in a climate chamber with controlled temperature and humidity. Accelerated stability tests were carried out at 40°C and 75% RH for 5 weeks. For spectrophotometric measurements, samples were taken at weekly intervals.

## METHODS AND INSTRUMENTATION

### UV-vis Analysis

The ASA hydrolysis was investigated using UV-Vis spectrophotometry. Due to spectral interferences, simultaneous determination of ASA and SA by direct UV spectrophotometry is not possible and determination by ‘zero crossing’ second-derivative was used [22].

#### Preparation of ASA and SA Solutions

Acetonitrile–formic acid (99:1 *V*/*V*) was prepared as a solvent mixture. Stock solutions at the concentration of 500 μg mL^−1^ for ASA and 100 μg mL^−1^ for SA were prepared separately. Working standard solutions were obtained by dilution of the respective stock solution. Series of solutions within the concentration range 25–250 μg mL^−1^ for ASA in the presence of SA (5 μg mL^−1^) and 5–50 μg mL^−1^ SA in the presence of ASA (150 μg mL^−1^) were used to prepare calibration curves.

#### Assay Procedure for Polymer/ASA Mixtures

During a 5-week experiment, 20 mg of each polymer/ASA mixture at weekly intervals was taken, transferred to 10 mL flask, and diluted with the solvent mixture to the volume mark. The mixtures were sonicated for 10 min and centrifuged (4000 rpm, 4 min). Clear supernatants were diluted tenfold with the solvent mixture.

Spectrophotometric analysis was carried out by means of UV-Vis Cary 100 Conc (Varian) spectrophotometer using 1 cm quartz cells. The zero-order absorption spectra of ASA and SA were recorded from 200 to 400 nm (using acetonitrile–formic acid 99:1 *V*/V as a blank) and converted to the second-derivate spectra. The absolute values of the derivate were measured at 292 nm for ASA and 328 nm for SA determination. Parameters of calibration curves for ASA and SA were shown in Table II.

**Table II:** Regression analysis of calculated contents of ASA and SA assay by second-derivate UV spectrophotometry

Type of drug	*λ*_max_	*R* ^2^	*b*	*a*
ASA in the presence of SA (5 μg mL^−1^)	292 nm	0,9971	3.0123 × 10^−4^	6.3103 × 10^−5^
SA in the presence of ASA (150 μg mL^−1^)	328 nm	0,9995	1.1749 × 10^−4^	1.0473 × 10^−4^

(*y* = *a* × *x* + *b*); *R*^2^ stands for a coefficient of determination

### DSC Analysis

The measured enthalpies of melting were normalized to the weight of dry samples and plotted against certain water *W*_*c*_ concentrations. The NFW contents were calculated from linear dependences (∆*H* = *f*(*W*_*c*_)), after extrapolation ∆*H* to 0.

The DSC experiments were performed in a nitrogen atmosphere with a flow rate of 50 mL min^−1^, using EXSTAR DSC 7020 apparatus (Hitachi Inc.) equipped with immersion cooler ULSP 90 (ULSP BV). The instrument was calibrated with 99.9999% indium and high purity Milli-Q water in accordance with the manufacturer’s instructions.

The measurements were carried out using the following thermal protocol: start at 20°C, cooling from 20°C to − 60°C at 3°C min^−1^, isothermal at − 60°C for 5 min, heating from − 60°C to 20°C at 3°C min^−1^. Sample pans were weight before and after each measurement to ensure that there is no loss of weight.

The interpretation of DSC curves was performed with Muse Measurement v 9.21 U software, using a T-Slice Analysis (Integral Tangential). Each sample was measured three times, and the obtained melting enthalpies were averaged. However, some of the samples were measured at least five times, and the %RSD was calculated based on the results.

## RESULTS AND DISCUSSION

**Fig. 1:**
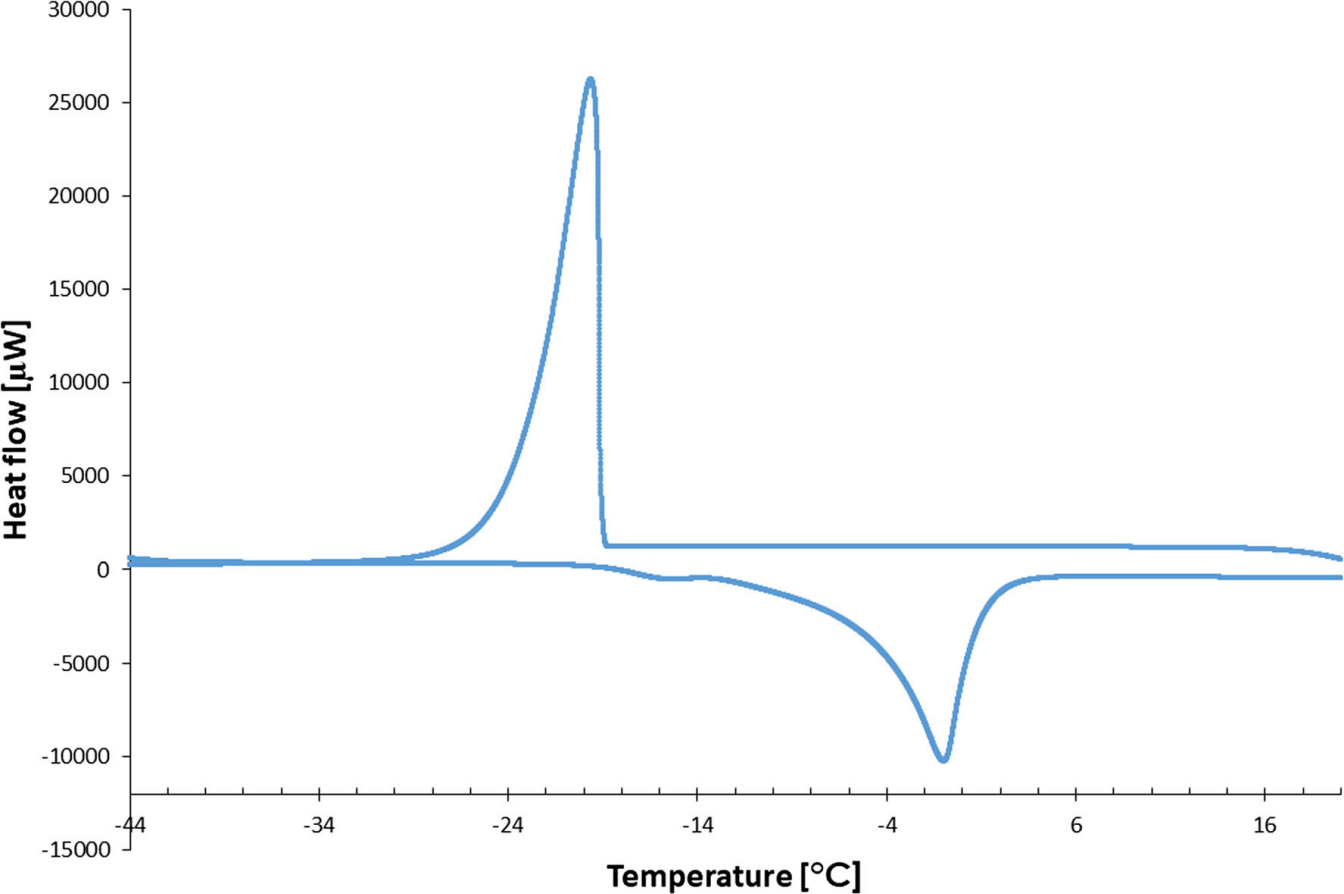
A representative DSC curve of crystallization and melting of hydroxypropyl methylcelluloses (3 cP) sample

A section of typical DSC curve showing crystallization and melting events, obtained from hydrated raw hydroxypropyl methylcelluloses 3 cP is shown in Fig. 1.

**Fig. 2:**
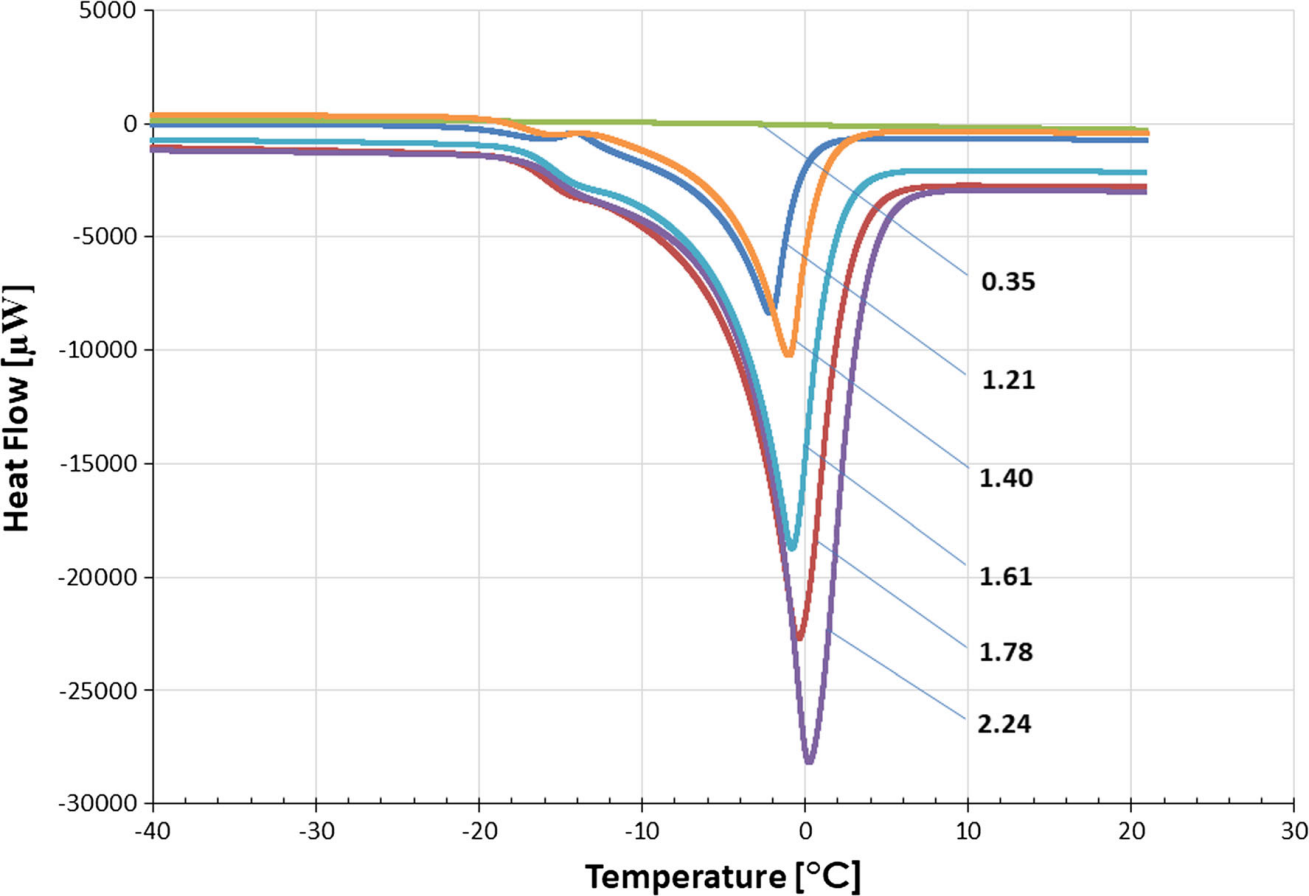
The stack of some shortened by a cooling part DSC melting curves, obtained from hydrated raw HPMC 3 cP samples; the numbers beside each curve are associated with water content *W*_*c*_

A series of representative DSC curves, obtained from hydrated raw HPMC 3 cP samples of *W*_*c*_ ranging from 0.35 to 2.24 g g^−1^ (shortened by cooling part), was collected as shown in Fig. 2. The first curve marked as 0.35 g g^−1^ can be considered a flat baseline because no endo- or exothermic events can be seen. It is possible that for concentrations of water under NFW content (0.49 g g^−1^ in this case—Table III), nearly all bound molecules are being strongly restrained by the hydroxyl groups of HPMC chains. When the content of water in the sample is slightly increased, the peak associated with crystallization is still absent while the melting peak can be seen. That phenomenon was also described in other publications [19, 21] and happens until *W*_*c*_ exceeds a certain amount. This amount varies in accordance with the structure of polysaccharide and accompanying ingredients. With higher contents of water, that exceed NFW, both crystallization and melting peaks can be seen, as it is shown in Fig. 1. Moreover, as the water concentration in the sample increases, the enthalpy value increases linearly, which was used in further studies.

**Table III:** Contents of non-freezing water NFW (in gram of water per 1 g of dry mass) determined for raw HPMC polysaccharides as well as for HPMC/acetylsalicylic acid and HPC/acetylsalicylic acid mixtures

Sample (viscosity [cP])	Parameters *a*; b	*R* ^2^	NFW [g g^−1^]
HPMC (3)	316.9; − 154.9	0.9959	0.49
HPMC (100000)	295.7; − 125.6	0.9980	0.42
LF (126)*	–	–	0.54*
MF (6300)*	–	–	0.54*
HPMC (3)/ASA	331.7; − 85.6	0.9945	0.21
HPMC (100000)/ASA	332.5; − 108.8	0.9960	0.33
LF (126)/ASA	297.9; − 37.5	0.9890	0.23
MF (6300)/ASA	341.3; − 95.4	0.9953	0.28

*For comparative purposes, similar contents of raw HPC/LF and HPC/MF, which were measured elsewhere [21], were also included (Δ*H* = *a* × *W*_*c*_ + *b*)

**Fig. 3:**
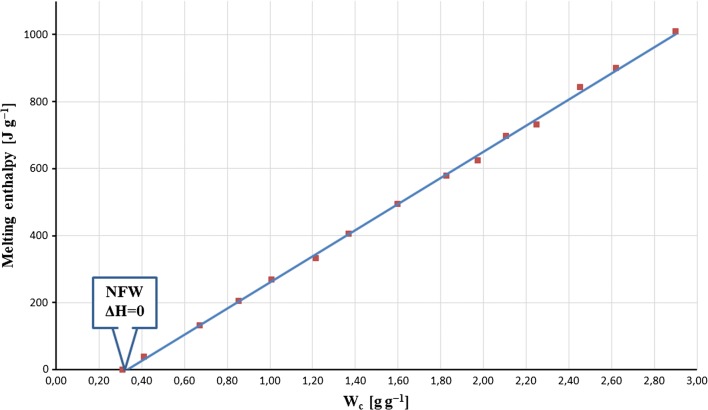
The relationship between determined enthalpies of melting and corresponding contents of water *W*_*c*_ represented linear dependences. The non-freezing water amounts were estimated after extrapolation to 0. The figure shows an example obtained for HPMC(100000)/ASA mixture

In order to calculate the non-freezing water contents, the measured enthalpies of melting of hydrates samples were normalized to the dry mass of polymer under study and then plotted against the corresponding water concentrations *W*_*c*_. A typical example of an obtained linear relationship (∆*H* = *f*(*W*_*c*_)) is shown in Fig. 3. The calculated coefficients of determination were high enough (Table III) to calculate the non-freezing water amounts based on extrapolation to 0 (∆*H* = 0).

Obtained values of NFW with parameters of linearization and coefficients of determination *R*^2^ for all studied samples are listed in Table III. The linear trend was maintained throughout the measuring range.

**Fig. 4:**
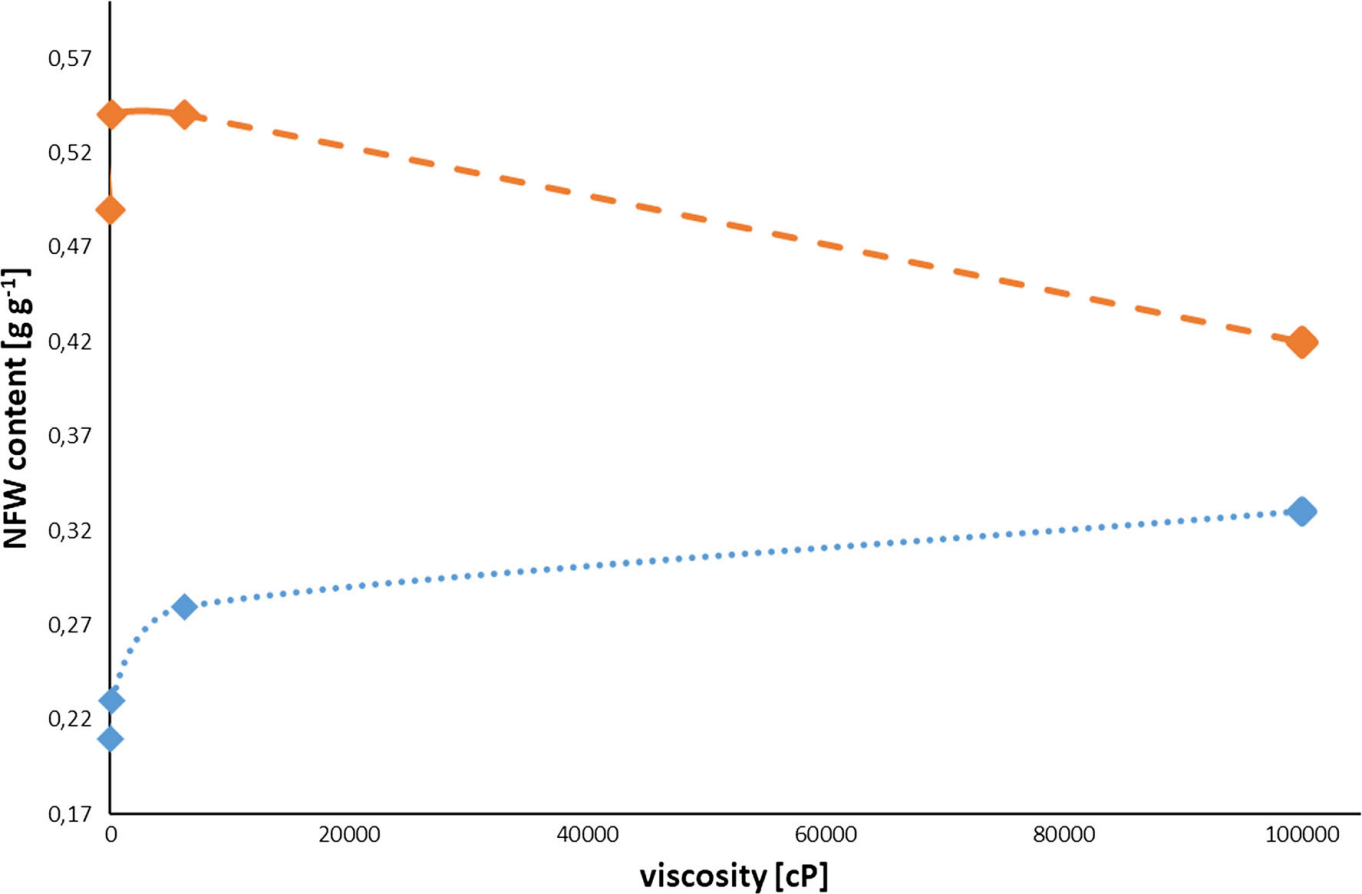
Relationship between NFW and viscosity obtained for raw of HPMC and HPC samples (dashed orange line) and for their mixtures with ASA (dotted blue line)

The content of non-freezing water calculated for all polysaccharides and their mixtures is ranging from 0.21 to 0.49 g g^−1^. The highest values were found for raw HPMCs of 3 cP and 100,000 cP and are 0.49 and 0.42 g g^−1^ respectively. For comparative purposes, similar measurements published elsewhere [21], and carried out for raw HPC type LF (126 cP) and MF (6300 cP) were presented. It was shown that NFW contents were 0.54 g g^−1^ for both samples (data marked with an asterisk). It can be concluded that a significant increase in viscosity is associated with a decrease in NFW content **(**Fig. 4**)**. Opposite conclusions can be drawn for polysaccharides/ASA mixtures, where the NFW values are smaller and equal to 0.21, 0.23, 0.28, and 0.33 g g^−1^ for HPMC/3 cP/ASA, HPC/LF/ASA, HPC/MF/ASA, and HPMC/100000 cP/ASA respectively. In that case, one can see that with increasing viscosity the NFW amounts are increasing as well (Fig. 4).

After 35 days of storing in a climatic chamber at 40°C and 75% RH, a decrease in ASA concentration was observed for all HPMC/ASA and HPC/ASA mixtures under study (Table IV).

**Table IV:** Percentage value of ASA in the initial quantity of the polysaccharide/ASA mixture after 5 weeks storing in a climatic chamber at 40°C and 75% RH compared with NFW contents

Polymer	NFW	ASA [%]
		week 5
HPMC 100 k [cP]	0,33	93.70
HPMC 3 [cP]	0,21	89.00
HPC/MF 6300 [cP]	0,28	85.95
HPC/LF 126 [cP]	0,23	85.05
ASA	–	100

As for other esters, hydrolysis of ASA was accompanied by a proportional increase in the salicylic acid content. In the HPC group, where viscosity differed by one order of magnitude, the ASA contents at the end of the experiment were comparable and calculated to 85.95% and 85.05% for HPC/MF and HPC/LF respectively. However, for the HPMC/ASA mixtures, the viscosity differed by five orders of magnitude, and ASA contents were calculated to 93.7% for 100,000 cP and 89.0% for 3 cP. This gives a difference of 4.7% compared to 0.9% found in the HPC group. On the other hand, one can see that within the group with the same spatial architecture (HPC or HPMC), the level of hydrolysis increases with decreasing viscosity (Table IV).

A similar relationship (variability within the group with the same spatial architecture) was observed by comparing the content of unhydrolyzed ASA to the NFW water contents (Table IV). It can be noticed, that with increasing NFW, the concentration of ASA that was not hydrolyzed is also increasing. In other words, the higher the NFW content, the greater the stability of acetylsalicylic acid within both HPC and HMPC group of mixtures.

## CONCLUSIONS

In this study, we examined the influence of viscosity and contents of non-freezing water on the stability of acetylsalicylic acid as a model moisture sensitive ingredient.

In the first part of the study, NFW contents were determined by differential scanning calorimetry. It was found that for both HPC and HPMC mixtures, the content of NFW increases with increasing viscosity, regardless of the chemical structure of polysaccharide.

The next part of the research consisted of determining the degree of hydrolytic degradation of ASA. This time, the influence of the chemical structure of the studied polymers appeared. In the group of HPC matrices, the viscosity differed by one order of magnitude between studied polymers. The ASA contents after 35 days were comparable and equal to about 85% of the initial amount in the sample, although NFW content was found to be slightly different. In the HPMC group, the viscosity values differed by five orders of magnitude between studied polymers. For those systems, the ASA hydrolysis was significantly lower. The highest content of ASA was found for the high-viscosity and the high NFW content matrix and was equal to 93.7%.

In summary, very high-viscosity HPMC formulations, which have a strong ability to bind water to the hydroxyl groups of hydroxypropyl methylcellulose chains (high NFW value), significantly reduce the contribution of moisture to hydrolysis reactions. Generally, less viscous systems increase the hydrolysis reaction rate, although the effect of NFW cannot be observed for HPC/MF and HPC/LF mixtures.
